# 3D rotational fluoroscopy for intraoperative clip control in patients with intracranial aneurysms – assessment of feasibility and image quality

**DOI:** 10.1186/s12880-016-0133-0

**Published:** 2016-04-19

**Authors:** Thomas Westermaier, Thomas Linsenmann, György A. Homola, Mario Loehr, Christian Stetter, Nadine Willner, Ralf-Ingo Ernestus, Laszlo Solymosi, Giles H. Vince

**Affiliations:** Department of Neurosurgery, University of Wuerzburg, Josef-Schneider-Str. 11, 97080 Wuerzburg, Germany; Department of Neuroradiology, University of Wuerzburg, Josef-Schneider-Str. 11, 97080 Wuerzburg, Germany; Abteilung für Neurochirurgie, Klinikum Klagenfurt, Feschnigstraße 11, 9020 Klagenfurt am Woerthersee, Austria

**Keywords:** Aneurysm surgery, Clip control, Intraoperative, Angiography, Vessel patency, 3D fluoroscopy, Contrast, Image quality, Post-processing

## Abstract

**Background:**

Mobile 3D fluoroscopes have become increasingly available in neurosurgical operating rooms. In this series, the image quality and value of intraoperative 3D fluoroscopy with intravenous contrast agent for the evaluation of aneurysm occlusion and vessel patency after clip placement was assessed in patients who underwent surgery for intracranial aneurysms.

**Materials and methods:**

Twelve patients were included in this retrospective analysis. Prior to surgery, a 360° rotational fluoroscopy scan was performed without contrast agent followed by another scan with 50 ml of intravenous iodine contrast agent. The image files of both scans were transferred to an Apple PowerMac® workstation, subtracted and reconstructed using OsiriX® free software. The procedure was repeated after clip placement. Both image sets were compared for assessment of aneurysm occlusion and vessel patency.

**Results:**

Image acquisition and contrast administration caused no adverse effects. Image quality was sufficient to follow the patency of the vessels distal to the clip. Metal artifacts reduce the assessability of the immediate vicinity of the clip. Precise image subtraction and post-processing can reduce metal artifacts and make the clip-site assessable and depict larger neck-remnants.

**Conclusion:**

This technique quickly supplies images at adequate quality to evaluate distal vessel patency after aneurysm clipping. Significant aneurysm remnants may be depicted as well. As it does not require visual control of all vessels that are supposed to be evaluated intraoperatively, this technique may be complementary to other intraoperative tools like indocyanine green videoangiography and micro-Doppler, especially for the assessment of larger aneurysms. At the momentary state of this technology, it cannot replace postoperative conventional angiography. However, 3D fluoroscopy and image post-processing are young technologies. Further technical developments are likely to result in improved image quality.

## Introduction

Cerebral angiography is the standard procedure for the diagnosis of cerebrovascular lesions [1, 2]. During surgery of intracranial aneurysms micro-Doppler and indocyanin green videoangiography (ICG-VA) are widely used to assess the occlusion of the aneurysm and patency of distal vessels [3, 4]. These techniques are easy and uncomplicated to use. However, a considerable disadvantage of both of these techniques is that for intraoperative assessment the vessels have to be accessible and visible. As a result, the approach and the operative manipulation have to be tailored to visualise the parent vessel, the aneurysm and the departing vessels after placement of a clip. Particularly in large aneurysms, this may result in more manipulation than otherwise necessary to access the aneurysm base and neck. Intraoperative angiography (IA), in turn, does not require this kind of wide exposition. However, it is more time-consuming and requires the positioning of an angiography catheter. We previously reported about the imaging of intracranial aneurysms before placement of an aneurysm clip [5] and the immediate use of this technique in the case of emergency in a patient suffering from intracerebral hemorrhage originating from a ruptured intracranial aneurysm [6]. This series evaluated if image acquisition by intraoperative 3D fluoroscopy with intravenous contrast agent combined with conventional post-processing software is, in principle, possible and supplies appropriate images for clip control after microsurgical clipping.

## Materials and methods

This article is the report of a retrospective analysis of intraoperative 3D fluoroscopic angiography in a series of 12 patients with intracranial aneurysms for the assessment of distal vessel patency and clip control after placement of an aneurysm clip. Prior to aneurysm surgery, the patients were offered intraoperative 3D fluoroscopic angiography with the explicit information that the primary target of the procedure was the assessment of distal vessel patency after placement of the clip. They were distinctly informed about the potential risks of the administration of an iodine contrast agent and about the radiation exposure. All patients had given informed consent to the procedure and the possible publication of the results in an anonymized form. The production of data, analysis and publication were approved by the ethics committee of the medical faculty of the Julius-Maximilians-University Wuerzburg, Germany (Reference 268/13).

### Inclusion criteria

Patients met the criteria for intraoperative 3D rotational fluoroscopic angiography if they had an intracranial aneurysm, were over 18 years of age, and if microsurgical clipping of the aneurysm was indicated. Exclusion criteria were a history of hyperthyroidism, an allergy against contrast agent, or serum creatinine concentrations over 100 μmol/l (1.2 mg/100 ml). 12 patients were included in this analysis. Aneurysm details and assessibility before and after clip placement are listed in Table 1.

**Table 1:** Aneurysm locations and visibility by intraoperative 3D fluoroscopy before and after clipping. Vessel patency was well assessable in the majority of cases after clip placement. Complete aneurysm occlusion, however, was not reliably assessable in most patients. In patient 11, a neck remnant of the MCA aneurysm and clip displacement from the ICA aneurysm were confirmed by DSA. (Acom = anterior communicating artery, Pcom = posterior communicating artery, MCA = middle cerebral artery, ICA = internal carotid artery, A. = artery, SAH = subarachnoid hemorrhage)

Patient number	Aneurysm (size)	Preoperative visibility	Assessment of vessel patentcy	Assessment of aneurysm/neck remnant
1	Acom, coiled (4 mm remnant), SAH	(+) coil artifact	+	(+) no remnant
	Pericallosal A. (3 mm), incidental	+	not treated	not treated
2	Right Pcom (7 mm), incidental	++	++	+ mild carotid stenosis
3	Acom (14 mm), incidental	++	+	-
4	Acom (8 mm), incidental	++	+	+ no remnant
	Left MCA (6 mm), incidental	++	+	+ no remnant
5	Left MCA (8 mm), incidental	++	++	+ no remnant
6	Right MCA (7 mm), incidental	++	++	+ no remnant
7	Left MCA (5 mm), SAH	++	++	++ no remnant
8	Right MCA (9 mm), SAH	++	+	+ no remnant
9	Left ICA bifurcation (5 mm), SAH	++	(+)	-
10	Left MCA (9 mm), incidental	++	++	+ neck remnant
11	Left MCA (9 mm), SAH	++	++	+ neck remnant
	Left ICA (4 mm)	++	++	+ clip displaced
12	left Pcom (11 mm), incidental	++	+	+ no remnant

### Patient positioning and image acquisition

All patients were under general anesthesia and had received a central venous catheter in the right jugular vein for infusions of fluid, medication and contrast agent prior to the surgical procedures. Arterial blood pressure was continuously monitored via an arterial line in the left radial artery. All patients of the present series were placed in a supine position with their head fixed in a carbon Mayfield clamp. Prior to surgery, baseline imaging was performed as follows: Positioning of the 3D fluoroscope (O-arm®, Medtronic GmbH, Meerbusch, Germany) was verified by biplanar fluoroscopy. Subsequently, a 360° rotational native fluoroscopy scan was performed. For image acquisition, the “high definition mode” of the O-arm® (digital flat panel detector 40 × 30 cm, camera resolution 2000 × 1500, reconstruction matrix 512 × 512 × 192) was applied requiring an image acquisition time of 24 s for the 360° gantry rotation followed by a reconstruction time of 24 s. Thereafter, a second, contrast-enhanced scan with identical fluoroscopy parameters was performed using 50 ml of iodine contrast agent (Imeron® 350, Bracco Imaging, Konstanz, Germany). During the second scan, 50 ml of contrast agent were injected manually via the central venous line over 25 s. To obtain selective contrast filling of the arterial phase, the 360° fluoroscopy scan was started with a delay of 12 s after the beginning of contrast injection. After placement of the clips, all metallic surgical devices and spatula that were potential causes of artifacts were removed and the imaging procedure was repeated. The image files (DICOM data) were transferred from the O-Arm® to an Apple Power Mac® workstation and post-processed using OsiriX® imaging free software (OsiriX 4.0, 32-bit version).

### Post-processing of DICOM data

Native and contrast-enhanced data sets were subtracted using OsiriX® software. From the subtracted data set, a reconstruction was produced (Volume Rendering Mode). Bony structures were virtually removed and brightness and intensity modified in order to selectively demonstrate the contrasted cerebral vessels.

### Evaluation of images

The images before and after placement of the clips were assessed for visibility of the aneurysms, accessibility of distal vessel patency, and complete aneurysm occlusion by two observers (C.S. and N.W.) blinded to the patients’ pathology using a four-grade scale: - not visible/assessable; (+) poorly visible/assessable; + sufficiently visible/assessable, details not distinguishable; ++ clearly visible/assessable, details distinguishable. The observers were allowed to use all means of image reconstruction of the OsiriX software for their evaluation including no –subtracted and subtracted images, 2D- and 3D images, maximum intensity projections and volume rendering reconstruction procedures. Examples of images of 2D projections in 3 planes and an Excel file with the observers’ ratings can be viewed under https://www.dropbox.com/sh/3qw11d2z7djidwl/AACFxalr1gIPTs7MDjRcspqla?dl=0 and https://www.dropbox.com/s/6db2yzuw0y3ty2m/Aneurysm%20assessment.xls?dl=0.

## Results

### Patient characteristics

Twelve patients were included in the analysis. As most patients with aneurysms in the posterior circulation are treated by endovascular coiling in our department, all patients included in this analysis had aneurysms in the anterior circulation. Details on patients and aneurysms are shown in Table 1.

### Adverse events

We observed no adverse cardiocirculatory or anaphylactic reaction during or after contrast injection. One patient with a middle cerebral artery (MCA) aneurysm suffered an intraoperative seizure during retraction of the temporal lobe, supposedly independent of the administration of contrast agent.

### Radiation dose

A “High Definition Mode” with a higher radiation dose was used in order to improve image quality and to slow down the movement of the X-ray generator. 2 rotational fluoroscopy scans to be able to perform an image subtraction to reduce artifacts. The procedure was repeated after occlusion of the aneurysm. Thus 4 rotational scans were performed with a total dose-length product (DLP) of 4 × 376.82 mGycm. This equals approximately 1–1.5 the DLP of a whole-brain CT [7, 8].

### Image quality

**Fig. 1:**
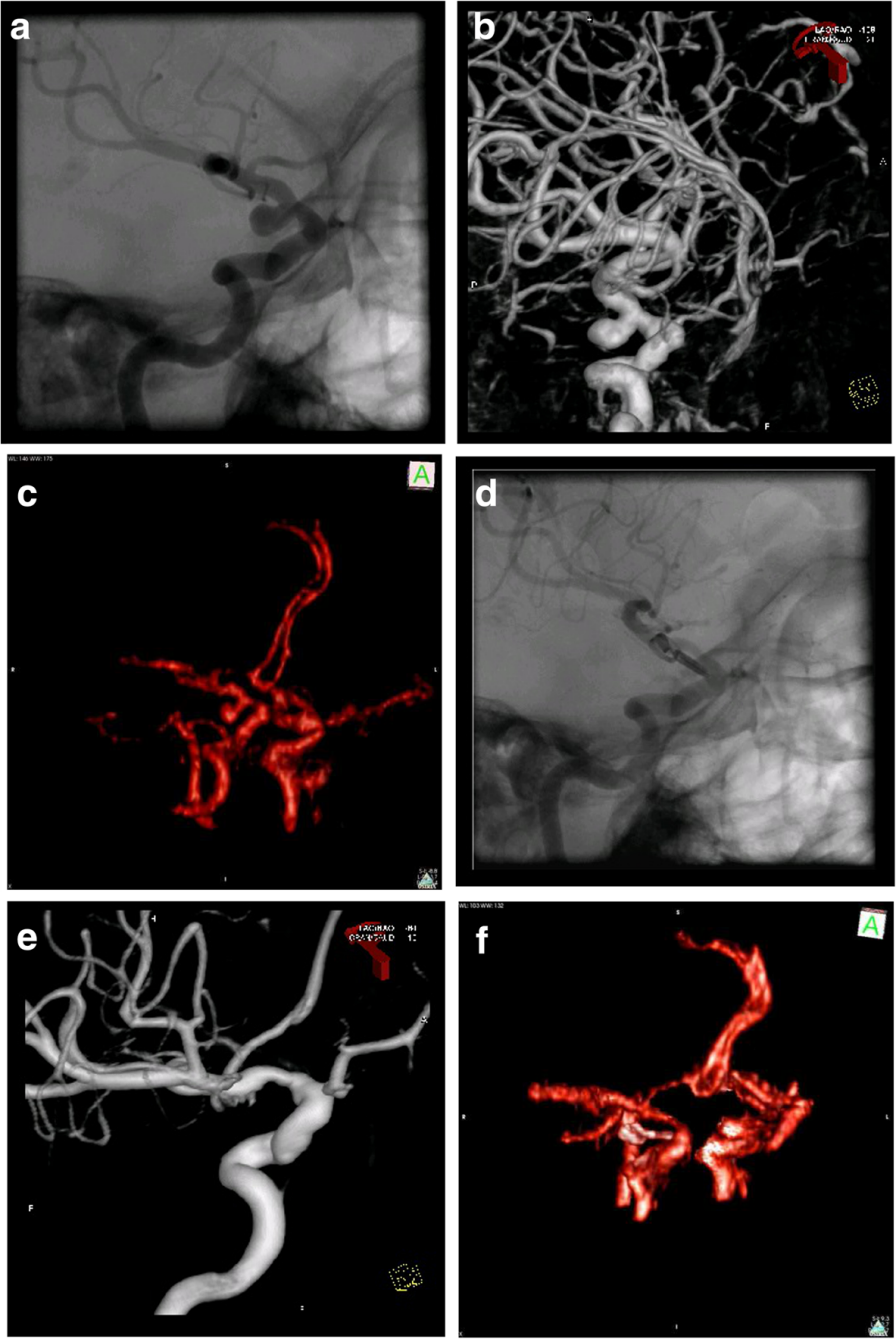
Preoperative DSA (**a**) with 3D reconstruction (**b**) and intraoperative 3D fluoroscopy after subtraction and reconstruction (**c**) before clip placement in a patient with a carotid artery aneurysm (case 2). **d**-**f** depict the respective projections after surgery. Postoperative DSA confirms a slight clip-induced narrowing of the ICA depicted by intraoperative 3D fluoroscopy

**Fig. 2:**
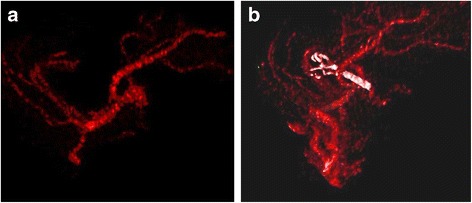
MCA aneurysm of case 4. MCA bifurcation before (**a**) and after (**b**) placement of the clip

**Fig. 3:**
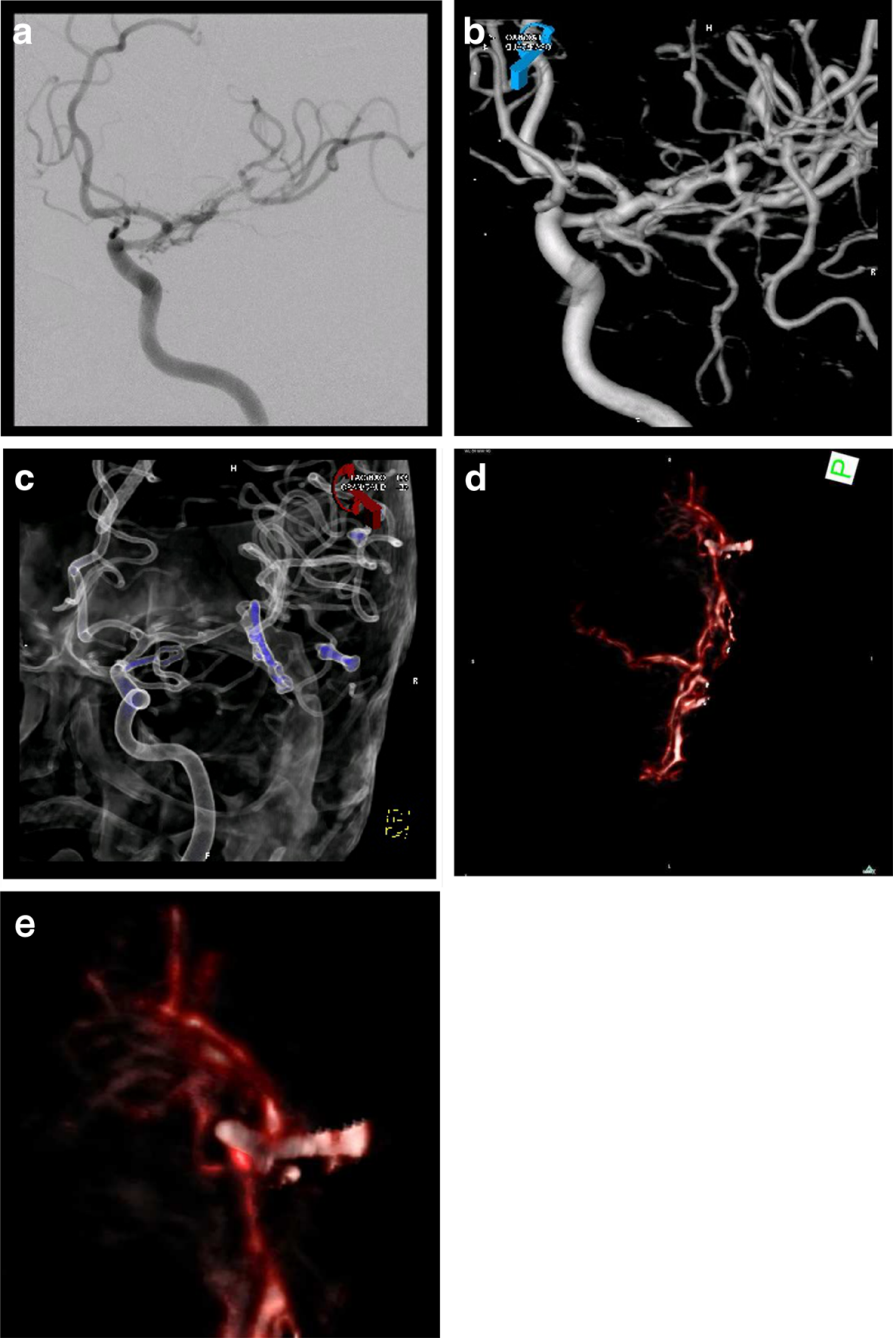
5 mm neck remnant after clipping of an MCA aneurysm in plain DSA images (**a**), 3D reconstructions (**b** and **c**) and intraoperative 3D fluoroscopy (**d** and **e**)

The observers’ ratings for preoperative visibility of aneurysms by 3D fluoroscopy and post-clip control of distal vessel patency and complete aneurysm occlusion is depicted in Table 1. In case 1, previous endovascular therapy of an aneurysm of the anterior communicating artery made the 4 mm neck remnant invisible to 3D fluoroscopy. Similarly, the occlusion of the aneurysm and the originating proximal anterior cerebral artery (ACA) branches could not be assessed. In contrast, the patency of the distal ACA, including a small (3 mm) aneurysm of the left pericallosal artery could be readily visualized. In all other patients of this series, the aneurysms were well assessable before clipping. It was observed that small vessels, especially close to the skull base, may not be visualized due to the occurrence of minor beam hardening artifacts and due to the limited image resolution of the original O-arm® images generated by the given image acquisition parameters. (see Fig. 1a-c). The patency of larger-aneurysm-carrying-vessels and their main branches (Fig. 2) was clearly visible after placement of the clip. Subsequently, a slight clip-induced narrowing of the internal carotid artery was visualized by intraoperative 3D fluoroscopy (Fig. 1f) and verified by postoperative digital subtraction angiography (DSA) (Fig. 1d and e). Similarly, the vasospasm-induced narrowing of the distal MCA trunk in case 11 which was most distinct close to the MCA bifurcation was depicted by DSA (Fig. 3a–c) and by intraoperative 3D fluoroscopy (Fig. 3d). After positioning of the clip, vessel patency distal to the clip was generally verifiable. In general, the exclusion of the aneurysm dome after clipping was also assessable. However, metal artifacts immediately surrounding the clip branches may remain in some cases in spite of image subtraction and made the verification of complete aneurysm occlusion not possible in most cases of this series. The presence of neck remnants may, therefore, remain undetected (Table 1). However, with precise image subtraction and post-processing, it is possible to depict larger aneurysm remnants (Fig. 3d).

## Discussion

The present case series shows that intraoperative imaging of cerebral vessels and assessment of aneurysm occlusion and vessel patency is possible using 3D rotational fluoroscopy and intravenous contrast agent with appropriate imaging parameters and post-processing software. After a first report on the use of intraoperative 3D rotational fluoroscopy and intraarterial contrast administration for the imaging of cerebral vessels in preparation for electrode implantation in epilepsy surgery [9, 10], we recently demonstrated that cerebral aneurysms can be depicted at adequate image quality using this imaging technology and intravenous contrast administration [5]. Apart from its reported usefulness for the diagnostic imaging of cerebral aneurysms in highly emergent cases [6], evaluating this method for intraoperative evaluation of clip-control and distal vessel patency is a further step in its clinical use. The fundamental advantage of angiographic techniques over “local” techniques like micro-Doppler and ICG-VA is that the vessels do not have to be completely exposed to gain visual control. This is of particular relevance in large aneurysms or after the placement of multiple clips. In both scenarios, the branching vessels may be concealed by the aneurysm or by the clips. In these cases, the application of micro-Doppler or ICG-VA requires a wider exposure which can be avoided by IA.

### Intraoperative clip assessment

Washington et al. and Caplan et al. have thoroughly evaluated the sensitivity of catheter-based IA and ICG-VA for the assessment of appropriate clip placement. In both studies, IA showed higher sensitivity than ICG-VA. Consequently, the role of IA as the gold standard for this purpose was underlined by the authors [11, 12]. Micro-Doppler, in turn, is highly sensitive for the assessment of vessel patency but neck remnants cannot be evaluated with this indirect, non-visual technique [3]. These drawbacks can be avoided using IA. Gantry tilts can supply almost any desired perspective. The addition of 3D rotational image acquisition adds a higher degree of image plasticity. Digital image subtraction has further improved image quality. Intraoperative rotational DSA is the ideal technology for this purpose but is limited to hybrid-operating rooms which are only available at a small number of neurosurgical centers. In addition, patients are exposed to a relatively high radiation dose by DSA and its intraoperative use is comparatively time-consuming. It is invasive as it requires the placement of an intraarterial catheter, which necessitates heparinization if placed before surgery or delays surgery if placed after clipping. Intravenous contrast administration, as performed in the present series, can be integrated in the surgical workflow without any further manipulations or invasive procedures.

Using advanced image processing technologies, computed tomography angiography (CTA) has been reported to have a similar diagnostic precision [13] regarding the presence and shape of an aneurysm and the vessel-anatomy for the primary screening for aneurysms [1]. Various series have been published that evaluated 3D fluoroscopic angiography and flat panel detector CT (FD-CT) for the diagnosis of a stenosis of intra- and extracranial vessels and ischemic stroke by intraarterial [14] and intravenous [15] contrast administration and found a good correlation with angiography findings [16]. These studies, however, addressed untreated vessel pathologies and did not have to deal with metal artifacts. Leng et al. used intraoperative FD-CT and intraarterial contrast agent for aneurysm imaging and post clip control in a hybrid operating suite. The authors found good accuracy before and after aneurysm clipping [17]. Intraoperative flat-panel detector 3D rotational angiography with intravenous contrast has, to our knowledge, not been evaluated for the assessment of clip-control after aneurysm surgery. It may be superior in terms of reducing metal artifacts due to the intraprocedural image subtraction. However, an assessment of this technique for its ability to detect aneurysm remnants still has to be conducted. Schnell and coworkers assessed the accuracy of ICG-VA and intraoperative CTA and CT-Perfusion (CTP) for post-clipping assessment. As ICG-VA supplies a good view of the local field, intraoperative CTA/CTP offer conclusive information on the distal vessel patency and tissue perfusion [18].

Mobile 3D-fluoroscopes can be positioned so that they do not interfere with the operative setting and – in contrast to IA and intraoperative CT – can be completely embedded into the surgical workflow. It is evident that this time-frame is important in case of tissue ischemia due to clip-induced vessel occlusion which is exactly the matter of intraoperative assessment. In this case, a significant delay of the intraoperative assessment of vessel patency will favour the development of infarction [19].

### Limitations

Our results demonstrate that this procedure can be easily performed and supplies images of acceptable quality concerning the assessment of the parent vessel and the perfusion of the branching vessels. Even smaller vessels are depicted with good quality in locations surrounded by brain tissue. However, quality seems to decrease in the immediate vicinity of the skull-base, most likely due to beam hardening artifacts, similar to CT [20]. The sensitivity of CT angiography to detect cerebral aneurysms has previously investigated and found to be low for aneurysms smaller than 4 mm [21]. Metal artifacts originating from clips and coils can further confine the diagnostic quality [22, 23]. Likewise, the limitations apply for 3D-fluoroscopy. Our images show that is similarly susceptible to metal artifacts as case 1 of our series affirms.

In spite of modern image subtraction techniques, the resolution of the baseline images acquired by the O-Arm® using the afore mentioned settings appears to have arrived at a limit at the present developmental stage of this technology. In unsubtracted images, star-like artifacts are produced by the clip. Similarly, coil artifacts in aneurysms which have previously been occluded by endovascular procedures cause metal artifacts. These can partly be dissolved by image subtraction. However, the immediate vicinity of the clip tends to be surrounded by a slim artifact seam which will conceal a small neck remnant, if present. This might be a problem of the automated image subtraction mode used for post-processing. This mode does not consider e.g. minimal movements of the fluoroscope during the rotational scan which may, however, be relevant in image subtraction and may lead to these artifacts. Using professional post-processing software, a further improvement of image subtraction and reconstruction can be expected. Slight movements of the O-Arm or minor movements of the patient due to mechanical ventilation may be further causes of blurring that will lead to a minor incongruence of image acquisition in spite of identical patient and fluoroscope positions. However, even if the incongruence is only small, it impedes the assessment of complete aneurysm occlusion. Significant movements of the fluoroscope can be prevented by a high definition image acquisition mode in which the image transducer moves at slower motor speed. The latter may be prevented by apnea during the 24 s of image acquisition. One further drawback for intraoperative use is the necessity to remove all metal instruments such as retractors and the need for a carbon head fixation to reduce artifacts.

Higher contrast doses, higher injection speed and a body height dependent injection protocol may further increase image quality. A faster image acquisition time may, combined with optimized injection algorithms, increase the arterial contrast load and selectively enhance the arterial phase of cerebral circulation. Finally, the images obtained by this technique are a summation of X-ray shots gathered during a 360° turn of the transducer and, thus, do not supply information about a possible delay of contrast filling, e.g. caused by a moderate vessel stenosis after placement of the clip. Since 180° image acquisition is sufficient to reconstruct a 3D image, this shortcoming could be solved by the assessment of consecutive 180° sections obtained during the 360° turn. By this way, an evaluation of a contrast delay could be possible.

Rotational fluoroscopy and processing techniques are evolving technologies. Paul and coworkers reported advanced post processing techniques superior to the ones used on this current series to enhance image quality and diagnostic accuracy in liver tumors using ultrafast cone-beam computed tomography [24, 25]. Further technical advances and improvements of image quality may be expected in the near future.

## Conclusion

Intraoperative 3D fluoroscopy with intravenous contrast administration can produce images useful for the assessment of vessel patency after aneurysm clipping. The presence of larger neck remnants can also be detected. Small aneurysm remnants and small vessels, especially those close to the skull-base may not be visible because the image resolution is not high enough with the parameters used for image acquisition in the present series. At the current state, this method of 3D fluoroscopic angiography is technically not mature, but advances can be expected in the near future. 3D rotational fluoroscopy, micro-Doppler and ICG-VA are not rival techniques but may be used complementarily during aneurysm surgery.

## Ethics approval and consent to participate

All patients had given informed consent to the procedure and the possible publication of the results in an anonymized form. The production of data, analysis and publication were approved by the ethics committee of the medical faculty of the Julius-Maximilians-University Wuerzburg, Germany (Reference 268/13).

## Consent for publication

Not applicable.

## Availability of data

Examples of 2D projections in 3 planes and an Excel file with the observers’ ratings can be viewed under https://www.dropbox.com/sh/3qw11d2z7djidwl/AACFxalr1gIPTs7MDjRcspqla?dl=0 and https://www.dropbox.com/s/6db2yzuw0y3ty2m/Aneurysm%20assessment.xls?dl=0.

## Abbreviations

ACA: anterior cerebral artery
CTA: computed tomography angiography
CTP: computed tomography perfusion imaging
DICOM: digital imaging and communications in medicine
DLP: dose length product
DSA: digital subtraction angiography
FD-CT: flat panel detector computed tomography
IA: intraoperative angiography
ICA: internal carotid artery
ICG: indocyanin green
ICG-VA: indicyanin green video angiography
MCA: middle cerebral artery
SAH: subarachnoid hemorrhage
VA: video angiography
